# Surface Water Processes Influencing Alterations in Pharmaceutical Chemical Composition following Wastewater Discharge into a Freshwater Estuary

**DOI:** 10.3390/toxics10110702

**Published:** 2022-11-17

**Authors:** Gregory Foster, Arion Leahigh, Thomas Huff

**Affiliations:** 1 Department of Chemistry and Biochemistry, Potomac Environmental Education and Research Center at the Potomac Science Center, George Mason University, Woodbridge, VA 22191, USA; 2 Shared Research Instrumentation Facility, George Mason University, Manassas, VA 20110, USA

**Keywords:** PPCPs, wastewater discharge, tidal freshwater potomac river, sediments, compositional alteration

## Abstract

The tidal freshwater Potomac River (TFPR) in the metropolitan Washington, DC region receives wastewater discharge from eight major wastewater treatment plants with the potential to impact water quality. A total of 85 pharmaceutical chemicals and personal care products (PPCPs) were analyzed in surface water and sediments using solid-phase extraction and QuEChERS, respectively, in conjunction with liquid-chromatography tandem mass spectrometry-multiple reaction monitoring quantitation (LC-MS/MS-MRM). A total of 52 PPCPs were quantified in both surface water and sediment. The most frequently quantified PPCPs in water included caffeine, fexofenadine, nicotine, sulfamethoxazole, hydrochlorothiazide, MDA, desvenlafaxine, and metoprolol ranging from 10 to 360 ng/L, and in sediment included diphenhydramine, escitalopram, desvenlafaxine, fexofenadine, sertraline and triclocarban ranging from 20 to 120 ng/g (dry weight). Comparisons of PPCP constituents in WTP discharge and adjacent surface water showed altered compositions reflecting dispersal and transformation processes acted quickly following contact of effluent with surface water. Although the PPCPs were present at their greatest concentrations in surface water near the WTP discharge zones, PPCP concentrations rapidly attenuated yielding mainstem TFPR concentrations relatively consistent along the freshwater reach of the tidal range in the estuary. The PPCP concentrations in sediment maximized in the tributary shoals, but also decreased in the mainstem TFPR similarly to surface water. Compositional analysis showed sorption to geosolids was the most important factor in the loss of PPCPs following WTP discharge in the tributary embayments.

## 1. Introduction

Although the presence of pharmaceutical chemicals and personal care products (PPCPs) in natural waters has allied concern regarding the effects of these micropollutants over the past two decades [1,2,3,4,5,6,7], PPCPs remain largely unregulated except for a few exceptions on USEPA’s Contaminant Candidate 4 list for drinking water [8]. Thus, a greater understanding of the sources, fate and effects of PPCPs in surface waters will likely promote more effective management of emissions into the aquatic environment along with broader protection of public and environmental health. Because of the large number of PPCPs registered in commerce in the USA, including over 4000 active ingredient pharmaceuticals alone [9], defining the most relevant constituents in the aquatic environment is of upmost importance. Of most interest are prescription drugs, illicit/recreational drugs, over the counter medications, and personal care products (e.g., UV-filters) found at parts-per-trillion concentrations in natural waters and fluvial sediments that approach or exceed ecological risk factors [10,11,12,13,14,15,16,17]. Risks have been identified for PPCPs such as antibiotics (sulfamethoxazole), endocrine disrupter chemicals (4-nonylphenol, 17β-estradiol and 17α-ethynyl estradiol), caffeine, and benzophenone-UV filters (4-hydroxybenzophenone) in the aquatic environment [18,19,20], and undoubtedly others are likely to be discovered. Beyond the identification of PPCPs in water bodies, tracking changes in chemical composition occurring in surface water following WTP discharge through dispersal and degradation, such as sorption, biotransformation and photolysis, is also critical to evaluating and modeling the health risks of PPCPs in the aquatic environment.

The Potomac River is the fourth largest river along the eastern coast of the USA and second largest tributary of Chesapeake Bay by annual discharge [21], highlighting its importance to local water quality in the Washington, DC region. The tidal Potomac flows through the Alluvial and Estuarine Physiographic sub-Province of the Atlantic Coastal Plain between the Fall Line and confluence with Chesapeake Bay. The fluvial-estuarine transition zone of the Potomac River includes a shallow estuarine system with numerous tributary embayments that support critical spawning habitats for many freshwater and anadromous fish species [22], along with serving as a popular recreational resource for over 5 million local residents. The tidal freshwater Potomac River (TFPR) in the metropolitan Washington, DC region is the upstream tidal reach of the Potomac River estuary and receives discharge from eight major (i.e., >100,000 m^3^/d discharge capacity) wastewater treatment plants (WTPs). In the case of the TFPR, Chesapeake Bay lies 170 km downstream of Washington, DC and is a critical downstream water quality impact consideration.

PPCPs undergo a variety of dispersal and transformation processes in the aquatic environment following WTP discharge into surface waters [23]. Dispersal includes dilution, sorption and volatilization. PPCPs undergo sorption to varying extents in geosolids through a variety of mechanisms [24], but volatilization is generally regarded as a minor phase distribution process because of the small magnitude of Henry’s law constants of most PPCPs [6]. The chemical transformations of PPCPs occur primarily through photolysis, hydrolysis and biotransformation [25] in water and/or sediment, whereupon fate processes may take place on the time scale of downstream transport in rivers and streams, acting in some cases immediately after discharge. Dispersal and transformation dynamics alter the chemical composition and bioavailability of PPCPs in surface waters. The objectives of our study were to track the spatial composition of PPCPs in the TFPR and show specific chemical alterations taking place during downstream transport immediately following WTP discharge. The overall study goal was to identify the most likely hydrogeochemical factors that account for PPCP alterations in chemical composition in the TFPR. A better understanding of fate processes in surface water transport will enhance the utility of long-term status and trends monitoring and assessment programs.

## 2. Materials and Methods

### 2.1. Study Area

The Mid-Atlantic Coastal Plain is characterized as gently inclined, ranging from flat to deeply incised, with alluvial deposits that span coarse to fine (i.e., sand to silt) in texture with abundant organic matter derived from commonly occurring marshes [26]. The TFPR extends ~60 km through the first tidal segment of the Potomac River estuary ranging from Chain Bridge (Washington, DC, USA) just below the river fall zone at Great Falls (MD, USA) downstream to Quantico (Quantico, VA, USA). The estuary is broad and shallow (average depth ~4 m) in the freshwater region with a shoreline featuring several tributary embayments. These shoals are somewhat protected from the full force of the flow of the main body of water and are highly diverse and productive in terms of fish species and submerged aquatic vegetation [22]. The WTPs of focus in this study all discharge into streams that flow into nearby embayments. The TFPR is bordered by the densest urban areas of Washington D.C. and its suburbs supporting 84% of the Potomac basin population, with an average population density of 8470 per km^2^. The TFPR has shown historically poor water quality with eutrophication and high turbidity in this region [27].

### 2.2. Sampling

**Figure 1:**
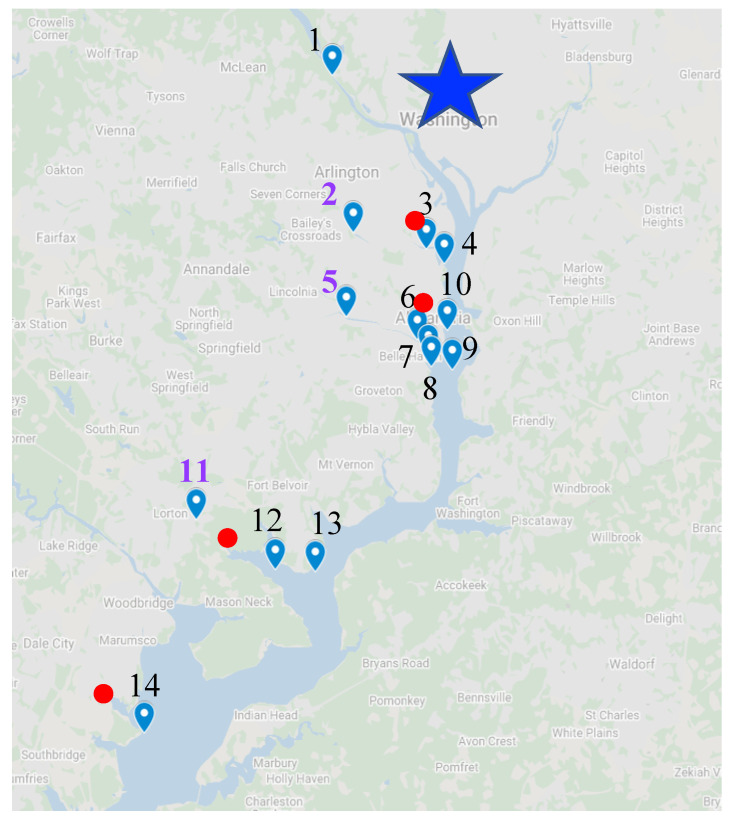
Sampling map of locations in the upland and tidal freshwater Potomac River watershed from Chain Bridge to Leesylvania State Park, VA. The city of Washington, D.C. is shown with a star. WTPs are shown as red dots and upstream reference sites are labeled with purple numbers on the map. Site names are 1, Potomac River at Chain Bridge; 2, Four Mile Run reference; 3, Four Mile Run near WTP; 4, Four Mile Run downstream; 5, Cameron Run reference; 6, Upper Hunting Creek near WTP; 7, Lower Hunting Creek downstream 1; 8, Lower Hunting Creek downstream 2; 9, Potomac River mainstem at Lower Hunting Creek; 10, Potomac River mainstem at Alexandria; 11, Pohick Creek reference; 12, Gunston Cove 1; 13, Potomac River mainstem at Gunston Cove; 14, Leesylvania State Park.

Surface water and surficial bottom sediment samples were collected from several locations throughout the mainstem TFPR along with several tributary embayments and upstream fluvial locations as reference sites (Figure 1 and Table S1). Chain Bridge was selected as the most upstream TFPR site since this location is at the beginning of the estuary and a short distance downstream of the Potomac River Fall Line. The WTP discharge-receiving areas of Cameron Run/Hunting Creek, Four Mile Run, and Gunston Cove were sampled with at least one site upstream of the WTP discharge, one site immediately downstream of the WTP discharge, and at least one additional site further downstream near entrance into the mainstem Potomac River. The Leesylvania State Park pier (site 14) was selected as the most downstream site. The features of the four WTPs associated with sampling in the TFPR (Table S2) serve four sewer-sheds in Northern Virginia region with some variations in population size. The most intensively sampled transect was Cameron Run/Hunting Creek/Potomac River, sites 5 through 9, ranging from above the WTP discharge zone of WTP 2 to the mainstem Potomac River.

Sampling was performed three to four times at each location on an approximate monthly basis through the period of May to September 2018 during ebb tide, employing a synoptic sampling approach within each of the tributary sub-regions having multiple sites (e.g., Hunting Creek, Four Mile Run, Gunston Cove). Surface water samples were obtained as grabs either onboard a 6 m skiff or by wading into shallow water using a submersible pump (Typhoon submersible pump, Max Flow 11 L/min, EnviroSupply & Service, Irvine, VA, USA). Each water sample (20 L) was collected at mid-depth. The typical water depth was 1.5 m. The water was collected in 20-L sealed stainless-steel kegs and transported to the Environmental Chemistry Laboratory at the Potomac Science Center, George Mason University. Upon return to the laboratory, the water samples were immediately filtered and stored at 7 °C prior to analytical processing within 48 h of collection. At each sampling site two additional 1-L water samples were collected in polypropylene bottles using the same pump method for the analysis of total suspended solids (TSS) at each site. All sample containers were pre-rinsed three times with sample water prior to filling.

Riverbed sediments were obtained onboard the skiff or via shoreline sampling coincident with water sampling when available fine-grained sediment was present (i.e., primarily silt-clay in composition). Upstream sites were often rocky or coarse-sand-bottomed and sediment was not obtained. Sediment was collected using a Petite Ponar grab tethered by hemp rope. The sediment obtained by the Ponar was taken aboard the boat or shore and carefully expelled into a stainless-steel tray minimizing disturbance to sediment. Approximately 10 g of the top 2 to 4 cm of the surficial layer was removed and placed directly into a pre-cleaned amber glass jar using a large stainless-steel spoon. The jar was sealed using a Teflon-lined lid and stored on ice for transportation. The samples were stored at −20 °C in the laboratory prior to further analytical processing.

### 2.3. Materials

Whatman^®^ glass microfiber filters, GF/F and GF/D, sizes 47 mm or 150 mm, were used for water filtration for small and large volume water samples, respectively, and were purchased from Sigma Aldrich (St. Louis, MO, USA). Oasis MAX and MCX, 6 mL cartridges (500 mg Sorbent per Cartridge, 60 μm particle size) were used in the extraction of all water samples and were purchased from Waters Corporation (Milford, MA, USA). QuEChERS (Agilent Technologies, Santa Clara, CA, USA) extraction and dispersive solid phase extraction (dSPE) salts and kits, used to process all sediment samples for LC-MS/MS analysis, were purchased from Agilent Technologies (Santa Clara, CA, USA). Acetonitrile and formic acid (FA), used to make the LC-MS/MS mobile phases, were purchased from Thermo Fisher Scientific (Waltham, MA, USA). Other bulk solvents used for analysis and supply preparation included methanol (MeOH), acetone, and ethyl acetate (EtOAc) were purchased from Thermo Fisher Scientific (Waltham, MA, USA). Milli-Q Type-1 water (MQW) was used to prepare LC-MS/MS mobile phase (Milli-Q Direct, EMD Millipore, Billerica, MA, USA).

### 2.4. Sample Preparation

The 20-L river water samples were initially filtered through a stacked combination of GF/D overlying GF/F glass fiber filters to clear suspended particles from water (Scheme S1). The filtered water was aliquoted into 1-L glass jars for subsequent extraction. Each surface water location was analyzed in triplicate via the aliquoted 1-L bottles. The filtered water was spiked with 50 to100 ng each of the internal and surrogate standards prior to extraction. An Oasis MAX cartridge (top) was coupled to a MCX cartridge using a tube adapter and the tandem set was attached to a Supelco vacuum manifold (Sigma Aldrich, St. Louis, MO, USA) on the outlet end and Teflon tubing (3 mm OD) to the sample bottle on the inlet end. Prior to extraction, the tandem MAX-MCX cartridge set was conditioned twice with 5 mL of 70:30 (*v*/*v*) MeOH:EtOAc, 5 mL of MeOH and 5 mL of MQW. The filtered samples were then loaded onto the cartridges via 3 mm (OD) Teflon tubing at a rate of 2 to 3 drops per second. Upon the conclusion of the extraction, the cartridges were washed twice with 95:5 (*v*/*v*) MQW:MeOH. The cartridges were aspirated on the manifold under vacuum for 30 min prior to elution. Following aspiration the MAX cartridges were eluted with 6 mL of 69:29:2 (*v*/*v*/*v*) MeOH:EtOAc:FA. The MCX cartridges were eluted with 6 mL of 67.5:27.5:5 (*v*/*v*/*v*) MeOH:EtOAc:NH_4_OH. The SPE extracts were combined in a 40 mL amber vial and reduced in volume to 0.5 mL using a TurboVap (Zymark Corp., Hopkinton, MA, USA) evaporator (employing dry N_2_ gas). The evaporated extracts were filtered using 25 mm dia. PDVF syringe filters attached to a 5 mL glass syringe during transfer to 1.5 mL amber glass autosampler vials.

Thawed sediment was pre-sieved (0.5 mm) to reduce large particle bias prior to characterization. In PPCP analysis, wet sediment (corresponding to 2 g of dry sediment) was spiked with internal and surrogate standards and extracted using a QuEChERS method [28,29] as summarized in Scheme S1. Briefly, 10 mL of acetonitrile + 10 mL of MQW were added to a 50 mL Falcon tube along with 6 g of anhydrous magnesium sulfate + 1.5 g of sodium acetate. The Falcon tubes were vortexed intermittently over 20 min while held on a shaker table. Following extraction, the tubes were centrifuged for 10 min at 2200 rpm and the acetonitrile (top) phase transferred to a 15-mL dSPE tubes containing 1.2 g of magnesium sulfate + 0.4 g of primary-secondary amine (PSA). The dSPE tubes were vortexed 4X over a 15 min period and centrifuged for 10 min at 2200 rpm, and the supernatant was transferred to a clean 40-mL amber glass vials for TurboVap (using dry N_2_ gas) solvent concentration to 0.5 mL. The evaporated solvent was filtered and transferred as described for water samples above to 2 mL autosampler vials. Each location where sediment was collected was extracted and analyzed in triplicate.

### 2.5. LC-MS/MS Analysis

PPCPs in water and sediment extracts were quantified using a Shimadzu Model 8050 tandem liquid chromatograph-mass spectrometer (LC-MS/MS) configured with a SIL-20ACXR autosampler (Columbia, MD, USA). The LC-MS/MS interface was operated in DUIS mode using both positive and negative ionization at a scan speed of 30,000 u/s at 0.1 u step size, coupled with polarity switching of 5 ms. LC-MS/MS separation of the PPCPs was performed using a 50 mm × 2.1 mm (id), 1.8 μm (dia) particle Force Biphenyl reversed-phase UHPLC column (Restek, Bellefonte, PA, USA) in conjunction with a raptor Biphenyl guard column, with a binary mobile phase consisting of MQW (solvent A), and acetonitrile (solvent B), both containing 0.1% formic acid as a phase modifier. Operating conditions for the LC-MS/MS are listed in Table S3.

LC-MS/MS identification and quantitation of the PPCPs was accomplished using MRM mode and included 3 MRM ions for each target chemical (with a few exceptions). The MRM ions were established for each PPCP through automated MRM optimization procedures following manual precursor ion identification using the full scan mode. The quantifier (primary) and qualifier (secondary and tertiary) product ions and the various quadrupole voltages for the PPCPs are compiled in Table S4. Quantitation was performed using a 10-point internal calibration standard (ranging from 0.05 to 250 ng/mL) based on the primary product MRM ion abundance for each PPCP relative to that of an associated internal standard. The retention times and qualifier MRM ions relative abundances were used to confirm the chemical identity of the PPCPs. Data analysis and quantitation was performed using LabSolutions software (ver. 5.91).

### 2.6. Quality Assurance

Surrogate spike recoveries (N = 33) for sulfamethoxazole-^13^C_6_, alprazolam-d_5_, and benzophenone-d_10_ were 62 ± 12%, 102 ± 10% and 80±15% in surface water, and 67 ± 9%, 108 ± 11% and 104 ± 17% in sediments, respectively. Matrix-spike recoveries were performed for 60 of the PPCPs in surface water (25 ng/L) and sediments (20 ng/g), including all those detected in this study, obtained from sites 6 and 7 (Figures S1 and S2). The quantitation limit (*QL*) for all the PPCPs ranged from 0.54 ng/L to 51 ng/L in water and 0.39 to 26 ng/g in sediment (Table S4) and were determined according to Equation (1) as

(1) $$QL=\frac{S_{y}\times 10}{m\times V_{s}\left(M_{s}\right)}$$

where *S_y_* is the regression standard deviation at the y-intercept, *m* is the slope of the calibration curve and *V_s_* is the sample volume (L) for water or *M_s_* is sample mass (*g*) for sediment. All autosampler vial volumes were adjusted to 1.0 mL prior to injection. Method blanks were prepared using MQW and clean sand for water and sediment, respectively. Field blanks were prepared from MQW or sand, placed in 20-L beverage kegs (water) and taken into the field. The water field blanks were recirculated from the pump to the beverage can for 10 min prior to sampling at the first location of each trip. The jar containing sand was opened, stirred with a steel spatula and resealed. No target chemicals were detected in the laboratory blanks. Only 6 target chemicals were found in water field blanks but at concentrations below the QL, and only caffeine was detected in water above the QL in 14% of all field blanks. The other compounds sporadically detected below QL concentrations in water field blanks included nicotine (14% frequency), sulfamethoxazole (4%), fexofenadine (12%), and carbamazepine (10%). No target chemicals were detected in any of the sand field blanks. Concentrations of the PPCPs in samples are expressed as ng/L for surface water and ng/g dry weight for sediments. For PPCP compositional analysis, concentrations are expressed in terms of mole fraction-PPCPs, which is defined as mol of a single PPCP divided by total mol of all PPCPs in the sample. Total PPCP concentrations in surface water are represented as S_33w_ PPCP based on those quantified in water and S_39s_ PPCP for those quantified in sediments. 

All glassware used for sample storage and preparation was cleaned by washing with soap, rinsing with Type-I-MQW and fired at 425 °C overnight to ignite any interfering organic residues on surfaces that may have interfered with quantitative analysis. All laboratory materials were made of glass, stainless steel, or Teflon to avoid minimize contamination. The Teflon materials were cleaned the same way as glass, but without firing. All non-glass items were rinsed with methanol and air dried before use.

### 2.7. Ancillary Measurements

Sediment moisture for the determination of sediment dry mass concentrations was determined by difference gravimetrically using 1 to 2 g of wet sediment with drying at 60 °C for 48 to 72 h until a constant mass was obtained. Flow for each of the tributaries and Potomac River on sampling days were obtained from the USGS WATSTOR database (https://www.USGS.gov accessed on 10 August 2020 ).

## 3. Results and Discussion

### PPCP Concentrations in Surface Water and Compositional Alteration

A total of 33 PPCPs (∑_33w_ PPCP) were quantified (≥QL) in surface water (ng/L) within the TFPR. The PPCP concentrations showed significant differences spatially (Kruskal–Wallis, *p* < 0.05), with the greatest median concentrations observed near the two WTP outfalls at sites 3 and 6 (Figure S3A). The lowest ∑_33w_ PPCP median concentrations occurred along the Potomac River mainstem or at the fluvial upsteam reference sites for the tributary embayments, which were located above the head of tide. The five mainstem TFPR sites (1,9,10, 13 and 14) showed significant differences in surface water concentrations (Kruskal–Wallis, *p* > 0.05), with site 14 being an outlier. However, when site 14 was removed the four remaining TFPR mainstem locations were not significantly different in median PPCP concentrations (Kruskal–Wallis, *p* < 0.05). Site 14 was clearly influenced by the presence of WTP 4 (Table S2) discharging into Neabsco Creek, indicating that although site 14 was selected at the end of a long pier into the main Potomac River channel located at Leesylvania State Park it was not indicative of the mainstem TFPR. Although the high-capacity WTPs significantly increased surface water concentrations of PPCPs in the immediate vicinity of the outfall zones in the embayment regions, with a downstream influence of ~1 km radius, the WTP effluents did not appear to elevate the concentrations of the PPCPs further downstream in the mainstem Potomac River along the entire length of the TFPR from Chain Bridge to Gunston Cove (site 13). The major WTPs showed a near-field influence in the TFPR overall in terms of ∑PPCP concentrations (Figure S3A).

Because caffeine and nicotine were prominent upstream constituents in this geographical region, these two chemicals were removed from ∑PPCP consideration to provide ∑_31w_PPCP (Figure S3B) as an alternative comparison. The spatial profiles and statistical relationships along the TFPR were similar among the two ∑PPCP groups. However, it is even more clear the ∑_31w_PPCPs in the discharge zone embayments dispersed to consistent baseline (median) concentrations of 50 to 80 ng/L throughout the mainstem TFPR during the time period sampled at ebb tide. Removal of caffeine and nicotine sharpened the hydrogeochemical perspective of this process.

The PPCP concentrations measured in the effluents of WTP 1 and WTP 2 are illustrated in Figures S4 and S5. There were 52 PPCPs quantified in the WTP effluents ranging in concentration from 3.7 ng/L (fentanyl) to 9900 ng/L (fexofenadine) in WPT 1 to 2.8 ng/L (fentanyl) to 10,000 ng/L (fexofenadine) in WTP 2. The PPCP composition and concentrations were similar between the two WTPs. The individual PPCP concentrations (log transformed) were strongly correlated between the two effluents (Spearman’s Rho = 0.861, *p* < 0.05), which is expected given the similar socio-demographics of the two sewersheds in Northern Virginia. The important implication is the PPCP compositional profiles were likely consistent among the major WTPs in this reach of the river in correspondence to these two WTPs.

Conversely, when the unmixed WTP effluent concentrations (also log transformed) for WPT 1 and WPT 2 were compared with nearby discharge zone PPCP concentrations (Spearman’s Rho) at sites 3 (Four Mile Run) and 6 (Upper Hunting Creek), respectively, only weak correlations were observed (Rho = 0.25 for site 3, *p* > 0.05; Rho = 0.38 for site 6, *p* > 0.05). The weak correlations were not caused simply by dilution of effluent, because some individual PPCPs dissipated or changed in relative abundance relative to their effluent profiles.

**Figure 2:**
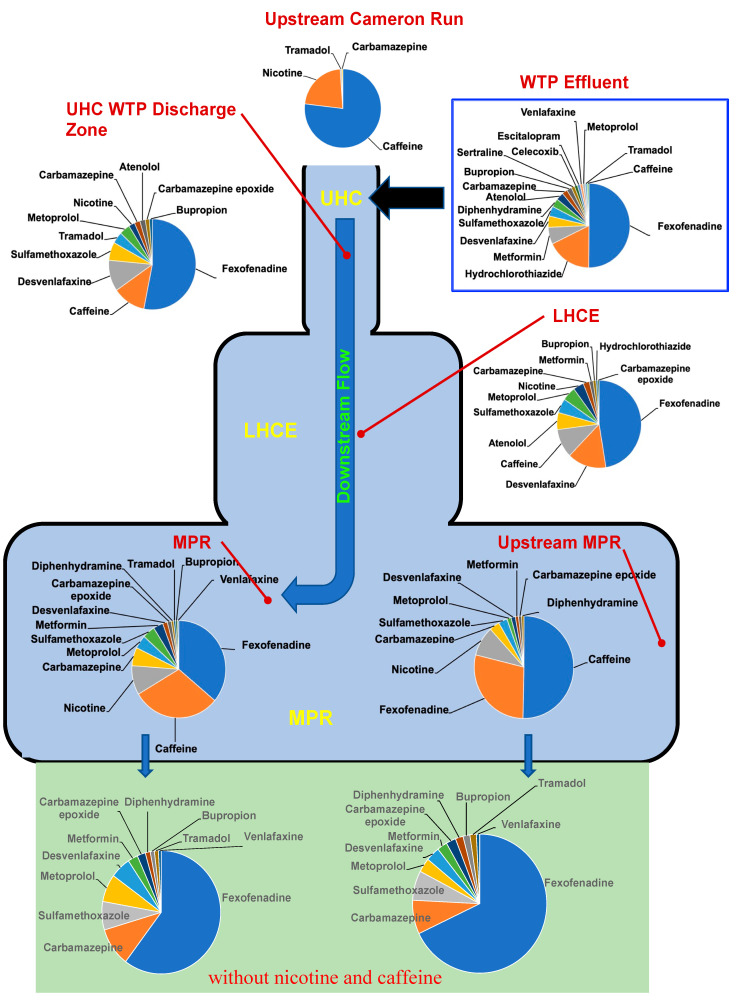
PPCP composition (% derived from ng/L) along downstream flow path of Cameron Run/Hunting Creek watershed into the tidal freshwater Potomac River. Bar charts represent 99% composition by mass. The black arrow denotes the outfall discharge point of WTP 2. (UHC, Upper Hunting Creek; LHCE, Lower Hunting Creek embayment; MPR, mainstem Potomac River.) Green shaded pie charts reflect composition comparisons without nicotine and caffeine included.

The Cameron Run/Hunting Creek watershed yielded the best trend of PPCP compositional alterations occurring in the downstream direction because of the extended transect at that location. The PPCP composition profiles demonstrated the typical upstream fluvial signature of caffeine and nicotine (Figure 2) as the predominant PPCPs (45 and 15 ng/L, respectively) and much lower concentrations of carbamazepine and tramadol (<5 ng/L). Because the Cameron Run site was above the head of tide, The PPCPs detected there were not contributed by WTP 2 and represented upstream fluvial sources. The Upper Hunting Creek WTP discharge zone (sampled within 50 m downstream of the outfall) showed many of the same PPCPs as in effluent but with a complete disappearance of escitalopram, sertraline, diphenhydramine, along with a considerable reduction of venlafaxine, metformin, atenolol and hydrochlorothiazide, well below a ~2-fold dilution factor upon mixing (a factor based on the time average ratio of volumetric flow rates of Cameron Run and WTP 2 effluent, Tables S2 and S5). Caffeine was a reliable WTP marker and illustrated the expected 2-fold dilution between WTP effluent and surface water in both Four Mile Run and Cameron Run (at sites 3 and 6). The individual PPCP concentrations quantitated in >50% of the surface water samples are shown in Figure S6 for sites 3 and 6. Furthermore, there was a considerable reduction in desvenlafaxine between Upper Hunting Creek and the mainstem TFPR through the Lower Hunting Creek embayment. The alteration in PPCP composition continued in the downstream direction through Lower Hunting Creek to the mainstem Potomac River along the site 6 to 9 transect (Figure 2). Caffeine and nicotine were minor constituents in WTP effluent, albeit at much higher concentrations than in upstream surface water. Caffeine and nicotine were, however, abundant in the mainstem TFPR. The contribution of both caffeine and nicotine from the Cameron Run upland watershed and WTP 2 was approximately equal on a first-order approximation. When caffeine and nicotine were removed from the compositional comparison for the two TFPR mainstem sites at this transect the remaining pharmaceutical composition at sites 9 (downstream of WTP 2) and 10 (upstream of WTP 2) were nearly identical (Figure 2). Thus, this illustrated the consistency of the PPCP composition (without caffeine and nicotine) following the tributary embayment input of a major WTP in the TFPR.

**Figure 3:**
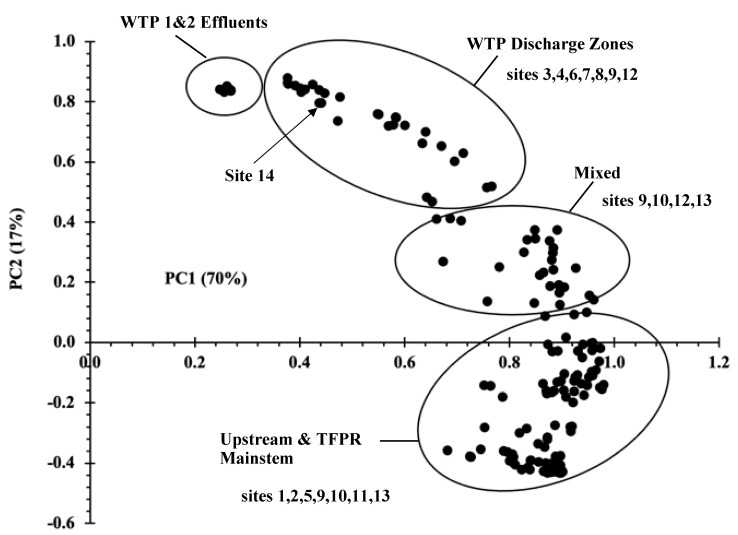
PCA loadings plot of PPCP composition across all tidal freshwater Potomac River sampling sites.

PPCP concentrations in water were converted to mole fraction-PPCP concentrations and analyzed by principal component analysis (PCA), revealing a continuum of changing composition from the upland streams to the mainstem TFPR. A spread of eigenvalues is apparent across a compositional arc beginning with the WTP effluents and culminating in the mainstem TFPR and upland locations. The PCA can be divided into 4 compositional segments (Figure 3), including (i) the WTP effluent from WTPs 1 and 2 (upper left quadrant in Figure 3), (ii) WTP discharge zone, (iii) transition mixture where the embayments flow into the mainstem TFPR and (iv) the mainstem TFPR itself in combination with the upland watersheds (lower right quadrant). The first two PCAs accounted for 87% of the compositional variability. The TFPR sites nearest the WTP outfalls showed a composition on the PCA in closest proximity to the effluents (e.g., sites 3, 4, 6–9, 12 and 14), but trending down and to the right. The opposite end of the PCA in the lower right quadrant included the sites directly upstream (above the head of tide) from the WTP outfalls (e.g., sites 2, 5, and 11) and some of the mainstem TFPR sites (e.g., sites 1, 10 and 13). The mixed zone included several sites that were primarily mainstem sites (e.g., sites 9, 10, and 13) that trended upward toward the discharge zone in the PCA. The PCA was very useful in proving that site 14 was impacted by WTP 4 effluent discharged near Leesynvania SP (site 14) because of its proximity to WTP effluents 1 and 2 in the loadings plot.

There were 39 PPCPs quantified in TFPR sediments (∑_39s_ PPCP), for which several PPCPs were unique to sediment. The ∑_39s_ PPCP concentrations (ng/g dwt) in sediment also varied spatially, with the greatest concentrations observed within a 1 km radius of WTP discharge (Figure S7). However, the distinct spatial feature of sediment PPCPs showed concentrations maximized in the embayments and not directly adjacent to the discharge points as was the case with surface water. The sediment spatial presence is again best exemplified by Hunting Creek (sites 6–8), whereby the maximum concentrations were found in the deposition zone of the Lower Hunting Creek (sites 7, 8). The deposition zone occurs where Lower Hunting Creek empties into its shoal and forms a bayhead delta. This sediment deposition zone clearly traps PPCPs emerging from WTP discharge undergoing downstream transport. Such a sedimentary process creates greater ecotoxicological risk in the benthic shoal community in the TFPR from PPCPs entering through the tributaries. However, the presence of relatively large ∑_39s_ PPCP concentrations in the Lower Hunting Creek shoal was a localized phenomenon because the mainstem TFPR sediments were much lower in concentration relative to the embayments.

The change in chemical composition of the PPCPs between effluent and nearby receiving waters shows that dispersal forces beyond dilution and reactivity acted on PPCPs rapidly following discharge. The most likely physical process acting on PPCPs immediately upon discharge was sorption in geosolids. PPCPs for the most part have low Henry’s law constants, such that air-water exchange is likely negligible. However, some PPCPs are highly particle-sorptive, and it has been shown previously that substantial PPCP fluxes occur into bed sediments within the discharge zone of Hunting Creek [30]. Degradative pathways such as biotransformation, hydrolysis and photolysis are also likely to alter the PPCP compositions in surface waters. For example, photolysis of hydrochlorothiazide, which was a predominant PPCP in our study, occurs rapidly in water with a half-life of 0.43 h [31]. As stated above, diphenhydramine, fexofenadine, sertraline, and escitalopram were observed in sediments in the TFPR, where affinity for sediment has also been reported by other studies [24,32,33,34]. Atenolol, metoprolol, caffeine and carbamazepine can be rapidly degraded by residual chlorine alone or in combination with UV-light [35]. Effluent has moderate residual chlorine concentrations, which is used as a disinfectant in tertiary treatment at WTPs. Ranitidine is rapidly transformed into a nitrosamine by-product in the presence of chlorine and UV-light [36]. Bupropion undergoes rapid hydrolytic degradation in aqueous solution at pH >5 to its most prominent degradation pathway that involves a hydroxide-catalyzed catalysis of the neutral base form [37]. The pH of receiving waters reported for the TFPR estuary have ranged from 6.8 to 7.8, depending on the season [38], promoting hydrolysis. All these examples above show how geochemical partitioning along with degradative forces act on PPCPs discharged into surface waters, contributing in many cases to rapid and extensive alterations in chemical composition.

**Figure 4:**
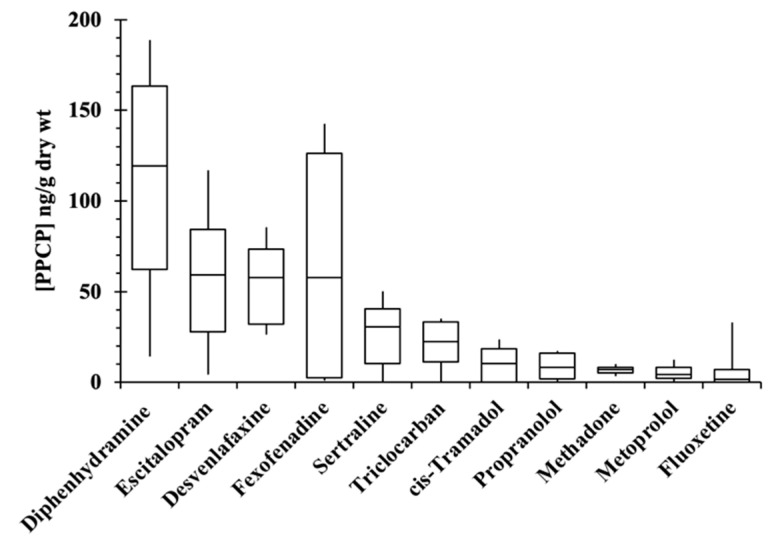
Box plot of sediment PPCP concentrations (ng/g dry wt) at site 8 for PPCPs quantified in >50% of the samples, where the sediment PPCP concentration were the maximum in the Cameron Run/Hunting Creek watershed. (Boxes represent 25th and 75th percentiles, midlines are the medians, and whiskers are the 5th and 95th percentiles).

There were 11 PPCPs detected at concentrations above the quantitation limit in sediments at ≥50% detection frequency. These included all the PPCPs shown in Figure 4 for site 8. All TFPR sediment sites showed PPCPs that were composed of subsets of these 11 constituents, except for Pohick Bay where triclocarban was quantified at >50% frequency in sediments. Further, the PPCPs detected in sediments showed no significant correlation (Spearman’s Rho = 0.15, *p* > 0.05) between log D_ow_ (n-octanol/water distribution constant at pH 7.4) and the measured conditional distribution constant, K_d-cond_ (K_d-cond_ = C_s_/C_w_ for PPCP concentrations quantified in sediments (C_s_) and surface water (C_w_) estimated in our study). The dynamic interaction of PPCPs with sediments is only partially explained by log D_ow_ because sorption to sediment occurs through electrostatic complexation mechanisms in addition to organic matter partitioning [39,40,41,42]. The K_d-cond_ estimates were often much larger than expected based upon the magnitude of log D_ow_, especially for PPCPs predicted to be positively charged (i.e., protonated) at ambient pH. Another possible reason for lack of correlation with log D_ow_ is because of rapid transformation that may be taking place in the environment (yielding low water concentrations). Furthermore, the sediment concentrations were not normalized to organic carbon levels because there was no observed correlation between K_d-cond_ and %TOC (Spearman’s Rho 0.10, *p* > 0.05). The %TOC in sediments is shown in Figure S8. It is generally assumed that organic micropollutants partition primarily into natural organic matter based on polarity and the (increasing) magnitude of D_ow_. Interactions of PPCPs between water and geosolids is a mixed complexation process, and the role neutral organic carbon plays in geochemical fate is likely not a dominant factor for many ionized PPCPs at ambient pH.

The PPCPs that contributed most of the variability in composition ranked according to the largest PCA eigenvalues are shown in Table 1. The list is divided between likely sorption dominant and reaction dominant processes. The sorption dominant factors are based upon those PPCPs found to be most enriched in TFPR sediment (Figure 4). Upon WTP discharge into the TFPR it is expected these PPCPs undergo rapid sorption to geosolids followed by deposition and incorporation into benthic sediment. As shown in Table 1, sorption accounted for the highest eigenvalue ranks and largest influence in altering PPCP concentrations in surface water transport. All five sorption-dominant PPCPs listed in Table 1 are positively charged conjugate acids of weak bases at ambient pH (~7.5), which form chemical complexes with negatively charged aluminosilicate geosolids [41]. The remaining PPCPs that were not observed to be particularly enriched in sediment are expected to undergo reactions primarily through photolysis (including hydrolysis) or biodegradation leading to a depletion of concentration in the TFPR. The most obvious case is hydrochlorothiazide with a reported photolysis half-life of 0.43 hr. Hydrochlorothiazide was a prominent constituent in WTP effluent but virtually non-existent in surface water. Alternatively, metoprolol showed an increase in abundance in surface water relative to effluent reflecting a lack of reactivity with only a minor degree of sorption. Venlafaxine is known to be demethylated to form desvenlafaxine as a major metabolite, which occurs primarily during wastewater treatment. Caffeine and nicotine are enriched in the TFPR predominantly from upstream sources and not WTP as described above.

## 4. Conclusions

Discharge from WTPs into the TFPR in the metropolitan Washington, DC region showed PPCP concentrations are elevated within a roughly 1 km radius downstream of the discharge point but attenuated downstream in the tributary embayments to a relatively consistent baseline concentrations in the mainstem TFPR. Further, changes in PPCP composition occurred over a short distance downstream following WTP discharge via sorption and reaction processes to yield a relatively stable PPCP composition in the mainstem TFPR. The effects of PPCPs are most likely to be limited to the tributary embayments in the vicinity of WTP discharge. The tributaries and associated embayments receiving discharge from high-capacity WTPs in Northern Virginia appeared to have only a minor influence on the total PPCP concentrations and PPCP composition in the mainstem TFPR during the times sampled.

## Figures and Tables

**Table 1:** Factors that account for PPCP compositional changes in TFPR surface water. Most important indicated by an X.

PPCP	Eigen Rank ^a^	Sorption Dominant	log D_ow_ (7.4) ^b^	Reaction Dominant	Half-life (h)	Reference/Other Comment
Sertraline	1	X	3.14			
Diphenhydramine	2	X	2.34			
Escitalopram	4	X	1.27			
Desvenlafaxine	7	X	0.89			
Fexofenadine	10	X	2.43			
Venlafaxine	3			X (Bio) ^c^	15	[43]
Nicotine	5					upstream source
Caffeine	6					upstream source
Hydrochlorothiazide	7			X (P) ^d^	0.43	[31]

^a^ Derived from the rank of largest PCA eigenvalues used in Figure 3; ^b^ n-octanol-water distribution coefficient at pH 7.4 predicted from EPI Suite [44]; ^c^ Bio is biodegradation; ^d^ P is photolysis.

## Data Availability

Original data used in our study are available upon request to the corresponding author.

## Supplementary Materials

The following supporting information can be downloaded at: https://www.mdpi.com/article/10.3390/toxics10110702/s1, Table S1: Details of sampling locations in the tidal freshwater Potomac River; Table S2: Pertinent Information on major wastewater treatment plants in the tidal freshwater Potomac River that were relevant to this study; Table S3: LC-MS/MS Instrument Parameters; Table S4: LC-MS/MS parameters of PPCPs subjected chemical analysis in this study; Table S5: Flow data for upstream locations on the day of each sampling event; Scheme S1: Water and sediment processing flow chart; Figure S1: Full target matrix spike (at 25 ng/L) recoveries in surface water (N = 7); Figure S2: Full target matrix spike (at 20 ng/g) recoveries in sediment using QuEChERS (N = 7); Figure S3: Box plots of of S_33w_PPCP concentrations in surface water for all PPCPs (**A**) and S_31w_PPCPs minus caffeine and nicotine (**B**). (Boxes are 25th and 75th percentiles; lines are the medians; red circles are the flow-weighted averages; and whiskers are the 10th and 90th percentiles.); Figure S4: Mean concentrations of PPCPs in effluent from WTP 1 (N = 3). Error bar is 1 SD of triplicate measurements; Figure S5: Mean concentrations of PPCPs in effluent from WTP 2 (N = 3). Error bar is 1 SD of triplicate measurements; Figure S6: Box plot of individual PPCP concentrations at Four Mile Run (site 3) near WTP 1 (**A**) and individual PPCP concentrations at Upper Hunting Creek (site 6) near WTP 2 (**B**). (Boxes represent 25th and 75th percentiles and whiskers 5th and 95th percentiles.) PPCPs are separated into high range and low range concentrations; Figure S7: Box plot S_39s_PPCP sediment samples in the tidal freshwater Potomac River. (Boxes are 25th and 75th percentiles; lines are the medians; and whiskers are the 10th and 90th percentiles.); Figure S8: Sediment %total organic carbon (%TOC) content (±1 SD) at sites in the tidal freshwater Potomac River. Site numbers correspond to Figure 1.
