# Supplementary material for: Surface Water Processes Influencing Alterations in Pharmaceutical Chemical Composition following Wastewater Discharge into a Freshwater Estuary
Source: Toxics. 2022 Nov 17;10(11):702. doi: 10.3390/toxics10110702 (PMC9696070; doi:10.3390/toxics10110702)
Supplement: Supplementary file 1 [file toxics-10-00702-s001.zip › toxics-1970929-supplementary.pdf]

toxics MDPI

Supplementary Materials: Surface Water Processes Influencing Alterations in Pharmaceutical Chemical Composition Following Wastewater Discharge into a Freshwater Estuary

Gregory Foster, Arion Leahigh and Thomas Huff

Table S1. Details of sampling locations in the tidal freshwater Potomac River.

Site Name	No. of Sites	Location	Site No.[a]	DD Coordinates	Sampling in 2018
Chain Bridge	1	Arlington, VA	1	38.9296, -77.11682	June, July, Sept
Upper Four Mile Run	1	Arlington, VA	2	38.84884, -77.10265	May, June, July, Sept
Four Mile Run	2	Arlington, VA	3,4	38.8405, -77.05262<br>38.83284, -77.04018	May, June, July, Sept
Upper Hunting Creek	1	Alexandria, VA	5	38.80543, -77.10747	May, June, July, Sept
Hunting Creek	5	Alexandria, VA	6-10	38.79367, -77.05887<br>38.78546, -77.05128<br>38.77958, -77.04911<br>38.77815, -77.0345<br>38.79839, -77.03847	May, June, July, Sept
Upper Gunston Cove	1	Lorton, VA	11	38.70129, -77.21021	May, June, July, Sept
Gunston Cove	2	Lorton, VA	12,13	38.67514, -77.15645<br>38.67399, -77.12894	May, June, July, Sept
Lower Potomac	1	Woodbridge, VA	14	38.5911, -77.24595	June, July, Sept

[a]Site number according to Figure 1 in text.

Table S2. Pertinent Information on major wastewater treatment plants in the tidal freshwater Potomac River that were relevant to this study.

WTP Name	Tributary of Discharge	Discharge Capacity (cubic meters per day)	Sewershed Population
WTP 1	Four Mile Run	175,000	~300,000
WTP 2	Hunting Creek	110,000	~315,000
WTP 3	Gunston Cove	300,000	~372,000
WTP 4	Neabsco Creek	109,000	~140,000

Table S3. LC-MS/MS Instrument Parameters.

Parameter	Operating Conditions
UHPLC Parameters	
UHPLC Flow Rate	0.40 mL/min
UHPLC Column Temp	40°C
UHPLC Gradient Elution Program	10% B at 0 min<br>50% to 95% B 0-6 min<br>100% B 6-7 min<br>100% to 30% B 7-9 min<br>10% B 9-10 min<br>10.01 min Off
MS Parameters	
Nebulizing Gas Flow	2 L/min
Heating Gas Flow	10 L/min
Drying Gas Flow	10 L/min
Interface Temperature	300°C

Table S4. LC-MS/MS parameters of PPCPs subjected chemical analysis in this study.

Chemical	Type	MRM[a]	RT[b] (min)	LOQ[c] Water (ng/L)	LOQ Sediment (ng/g dw)
Caffeine 13C3 IS	ISTD[a]	198.10>140.20	2.288		
Diazepam D5 IS	ISTD	290.10>198.20	6.361		
Sulfomethazine 13C6	ISTD	285.10>186.10	3.103		
Hydrocodone D6 SS	SSTD	306.20>202.15	2.477		
Desethylatrazine 13C3 SS	SSTD	191.10>149.20	2.888		
Sulfomethoxazole 13C6	SSTD[b]	260.10>162.10	3.789		
Ethyl Paraben 13C6 SS	SSTD	173.20>101.20	4.359		
Norsertraline 13C6	SSTD	281.00>159.05	5.675		
Alprazolam D5	SSTD	314.10>286.15	5.793		
Benzophenone D10 SS	SSTD	193.20>110.20	6.836		
Nicotine	Target[c]	163.30>130.30	0.454	1.9	2.1
trans-3′-Hydroxycotinine	Target	193.30>80.25	0.449	7.2	5.1
Acyclovir	Target	226.30>152.30	0.454	21	12
Cimetidine	Target	253.30>95.15	1.127	12	6.2
Cotinine	Target	177.30>80.20	0.458	6.8	3.0
Albuterol	Target	240.40>148.20	0.923	2.2	1.5
Atenolol	Target	267.30>145.25	1.024	5.7	2.9
Ranitidine	Target	315.30>176.25	0.463	8.9	7.3
Azithromycin	Target	591.50>116.10	2.146	3.5	1.7
Gabapentin	Target	172.40>154.30	1.506	11	4.5
Morphine	Target	286.40>152.20	0.933	19	9.4
Oxymorphone	Target	302.30>284.15	1.073	11	7.6
Clonidine	Target	230.20>44.20	1.784	12	6.2
2-Hydroxy Ibuprofen	Target	221.30>180.25	1.926	24	11
Hydromorphone	Target	286.30>185.20	1.000	16	6.7
Nadolol	Target	310.40>254.35	2.141	5.0	4.9
Caffeine	Target	195.30>138.25	2.284	15	7.9
Sulfathiazole	Target	256.20>92.10	2.457	6.5	4.7
Aspartame	Target	295.30>120.35	2.392	10	5.1
Penicillin G	Target	335.30>289.15	2.384	33	14
Amphetamine	Target	136.10>65.15	1.731	2.9	1.3
Methamphetamine	Target	150.00>91.20	2.001	3.1	2.7
Triamterene	Target	254.30>237.20	2.605	1.8	0.92
Naloxone	Target	328.40>310.20	1.888	1.4	6.1
MDA[d]	Target	180.40>163.25	1.939	4.9	5.5
Codeine	Target	300.30>165.30	2.005	4.1	2.6
Ciprofloxacin	Target	332.30>314.20	2.903	26	17
Phentermine	Target	150.00>91.20	1.983	8.9	3.8
Metoprolol	Target	268.40>116.20	2.949	6.4	3.2
Sulfamethazine	Target	279.30>186.20	3.107	6.2	3.7
Naltrexone	Target	342.40>324.20	2.311	1.4	2.7
MDMA[e]	Target	194.40>163.35	2.319	6.1	12
Enrofloxacin	Target	360.30>316.40	3.219	22	14
Formoterol	Target	345.40>149.30	3.301	34	12
Atrazine Mercapturate	Target	343.30>214.25	3.324	8.0	6.9
Hydrocodone	Target	300.30>199.20	2.486	12	6.7
cis-Tramadol	Target	264.40>58.20	3.132	5.2	2.6
Desvenlafaxine	Target	264.40>58.20	2.656	2.0	1.0
Bupropion	Target	240.30>184.20	3.646	2.8	1.4
Sulfamethoxazole	Target	254.30>92.10	3.781	8.3	4.2
Enalapril	Target	377.40>234.20	4.235	9.9	7.1
Propranolol	Target	260.30>116.20	4.316	2.4	1.1
Meperidine	Target	248.40>174.20	3.536	8.4	5.9

Sulfadimethoxine	Target	311.30>156.25	4.518	5.9	4.8
Dextromethorphan	Target	272.40>215.25	4.549	0.91	0.46
Sulfaquinoxaline	Target	301.20>92.10	4.638	4.2	3.1
Venlafaxine	Target	278.40>58.25	3.812	0.94	0.47
Diphenhydramine	Target	256.30>167.20	4.701	3.5	1.8
Diltiazem	Target	415.30>178.20	5.137	0.52	0.26
10,11-Carbamazepine Epoxide	Target	253.30>180.30	4.293	13	6.4
Promethazine	Target	285.30>86.20	5.169	1.7	0.96
Propoxyphene	Target	340.40>58.20	5.512	7.5	3.8
Fentanyl	Target	337.40>188.40	5.25	2.1	1.0
Verapamil	Target	455.40>165.30	5.679	10	5.1
Escitalopram	Target	325.40>109.10	4.806	51	26
Benztropine	Target	308.40>167.35	5.758	4.1	0.45
Buprenorphine	Target	468.50>396.30	5.800	4.8	4.6
Fexofenadine	Target	502.40>466.40	5.773	4.5	2.3
Carbamazepine	Target	237.30>194.25	5.059	5.8	2.9
Loratadine	Target	383.30>337.15	6.019	9.7	3.5
Naproxen	Target	185.30>170.20	6.101	27	14
Oxazepam	Target	287.20>241.10	5.295	0.75	0.39
Paroxetine	Target	330.30>192.40	5.289	3.6	0.96
Fluoxetine	Target	310.20>44.25	5.502	2.0	1.0
Nordiazepam	Target	271.20>140.25	5.365	13	5.2
Bezafibrate	Target	362.30>139.20	6.229	2.1	0.88
Nitrazepam	Target	282.30>236.20	5.332	2.4	0.45
Lorazepam	Target	321.30>275.05	5.427	15	7.3
Budesonide	Target	431.40>413.30	6.392	26	9.4
Nortriptyline	Target	264.30>233.25	5.514	1.7	0.88
Amitriptyline	Target	278.40>233.30	5.662	2.7	1.5
Methadone	Target	310.40>265.25	5.818	0.54	0.27
Clonazepam	Target	316.30>270.10	6.200	3.3	7.4
Alprazolam	Target	309.30>281.15	5.815	0.61	0.50
Sertraline	Target	306.20>159.10	5.885	21	11
Temazepam	Target	301.20>255.20	6.070	6.0	3.0
Diazepam	Target	285.30>193.25	6.417	1.8	0.81
Atorvastatin	Target	559.30>250.00	7.194	0.95	0.48
Triclocarban	Target	315.20>127.10	7.374	0.55	0.38
Lisinopril	Target	406.40>84.25	7.474	1.5	2.4
Tetracycline	Target	445.20>341.10	7.789	39	56
Hydrochlorothiazide	Target	296.20>205.20	2.171	4.8	1.9
Furosemide	Target	329.00>285.25	5.141	3.9	4.5
Glipizide	Target	444.20>319.25	5.974	9.7	3.0
Warfarin	Target	307.10>161.25	6.644	1.1	0.54
Diclofenac	Target	293.90>250.05	7.139	29	15
Celecoxib	Target	380.10>316.30	7.222	2.2	1.0

[a]MRM, multiple reaction monitoring ions.
ITSD is internal injection standard.
[b]SSTD is surrogate standard.
[c]Target is unknown chemical.
MRM indicates precursor ion>1° MRM ion.
RT is UHPLC retention time in minutes.
[d]3,4-Methylenedioxyamphetamine.
[e]3,4-Methylenedioxy-N-ethylamphetamine.

Table S5. Flow data for upstream locations on the day of each sampling event.

Sampling Location	Sampling Trip	Daily Average Flow (m^3/s)	Longterm Historical Avergage Flow (m^3/s)	Days since previous storm event
Potomac River-Little Falls	May	129	328 (89 years of record)	0
	June	1097		3
	July	372		13
Cameron Run	May	0.212	1.16 (56 years of record)	5
	June	0.416		4
	July	0.151		18
	Sept	0.852		4
Accotink Cr	May	0.263	0.75 (71 years of record)	12
	June	0.759		2
	July	0.430		11
	Sept	0.145		3
Four Mile Run	May	1.79	0.88 (44 years of record)	9
	June	0.96		5
	July	1.80		11
	Sept	0.81		3

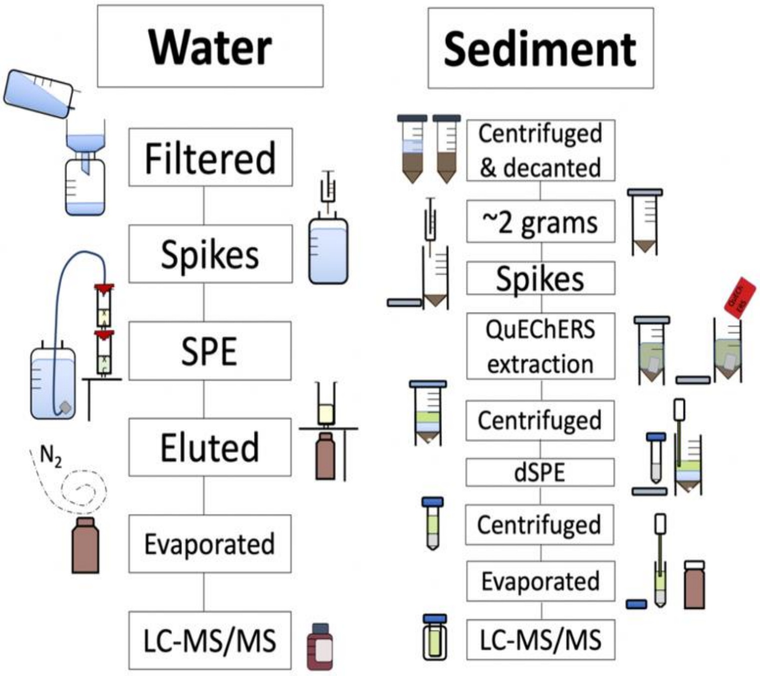

Scheme S1. Water and sediment processing flow chart (courtesy of Lisa McAnulty, George Mason University, Potomac Science Center).

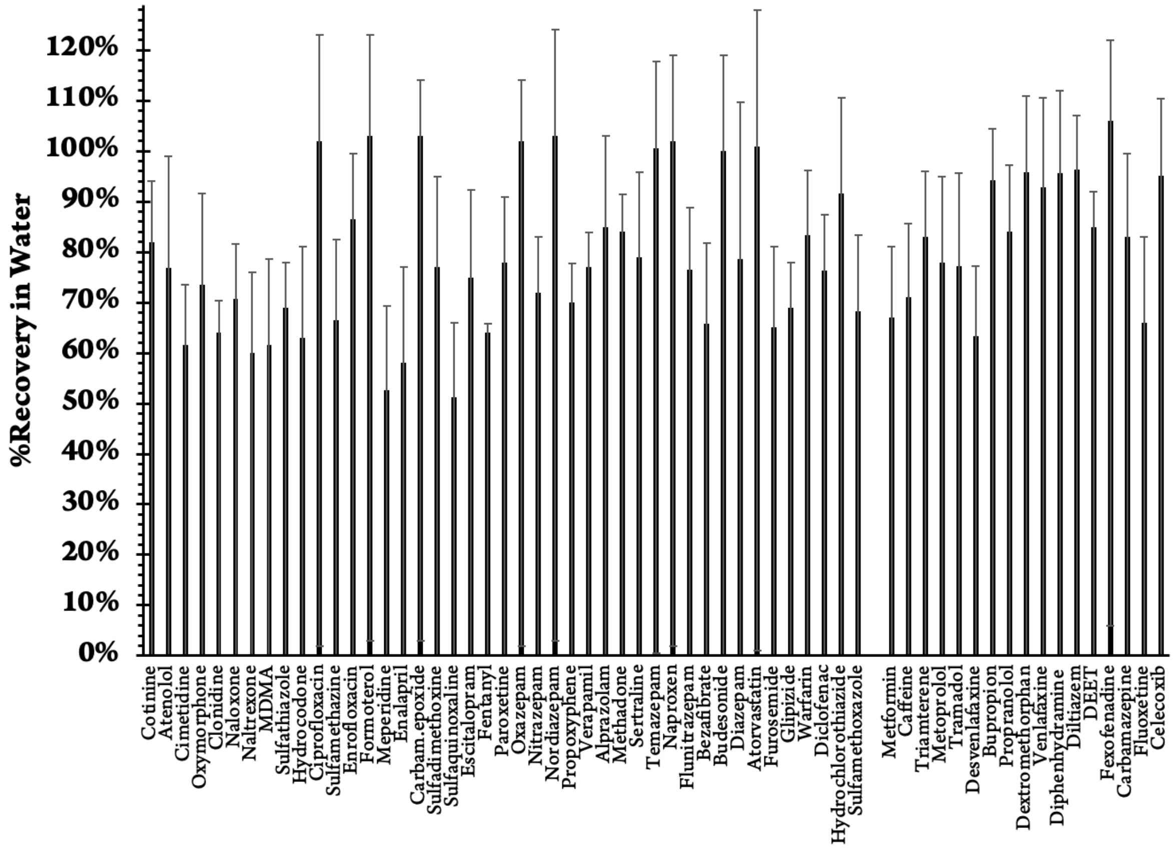

Figure S1. Full target matrix spike (at 25 ng/L) recoveries in surface water (N = 7).

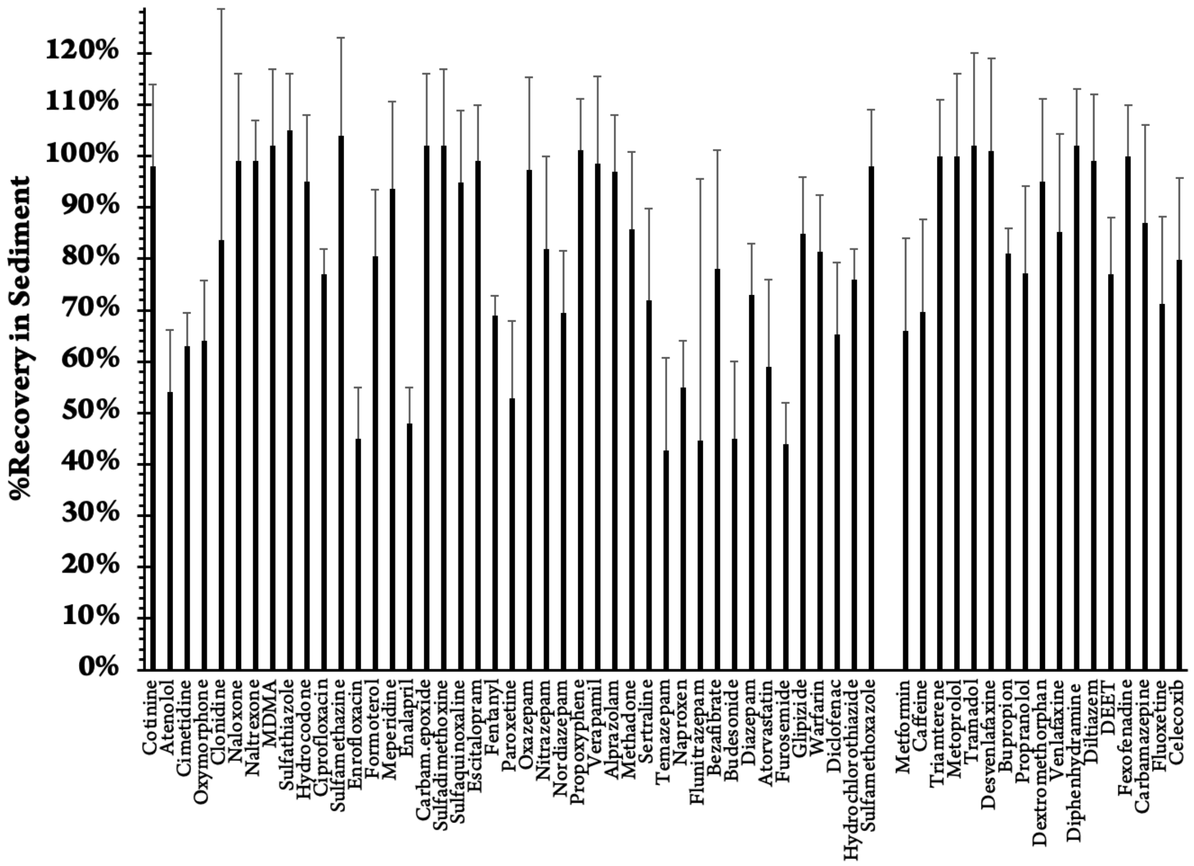

Figure S2. Full target matrix spike (at 20 ng/g) recoveries in sediment using QuEChERS (N = 7).

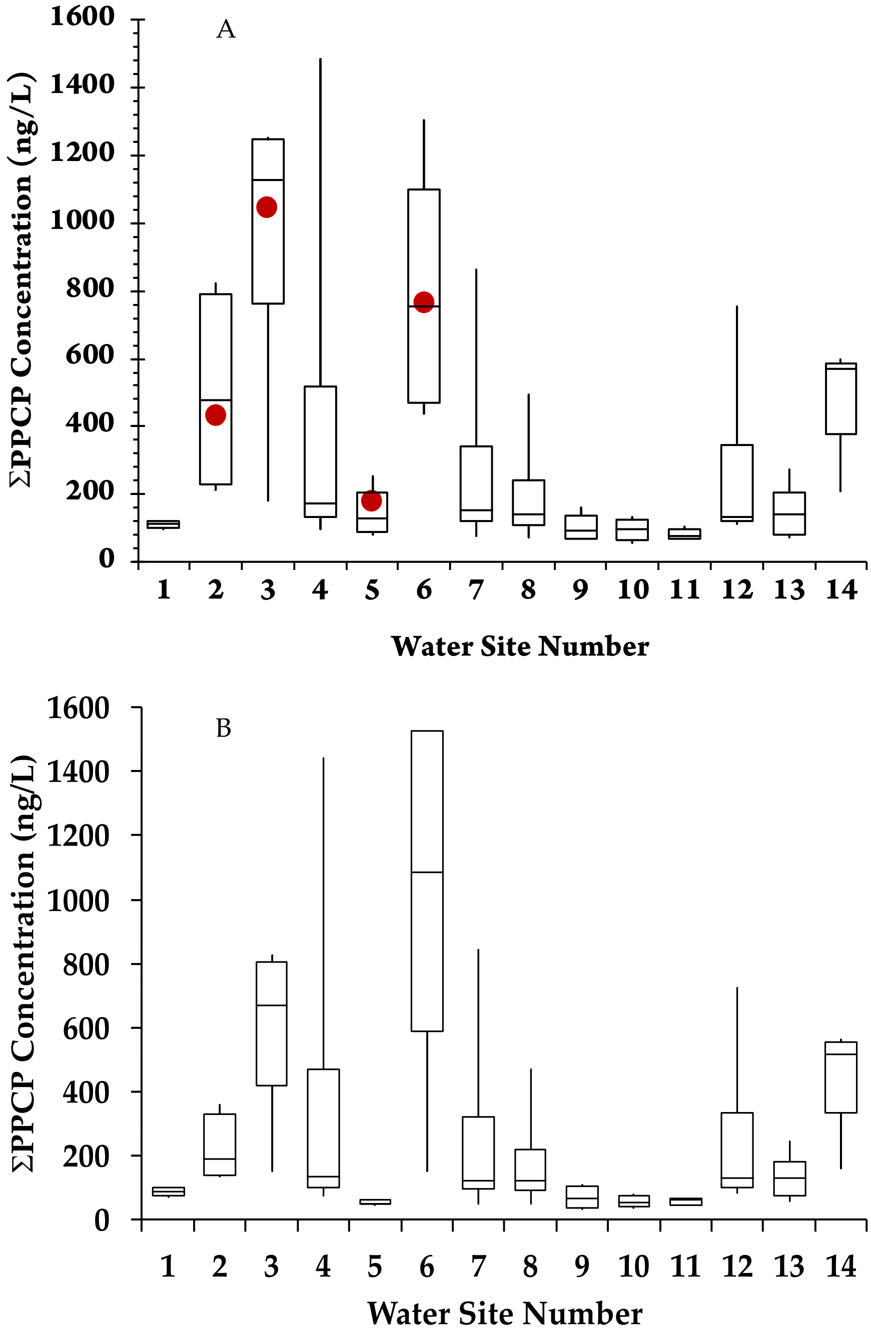

Figure S3. Box plots of of Σ_{33w}PPCP concentrations in surface water for all PPCPs (A) and Σ_{31w}PPCPs minus caffeine and nicotine (B). (Boxes are 25th and 75th percentiles; lines are the medians; red circles are the flow-weighted averages; and whiskers are the 10th and 90th percentiles.).

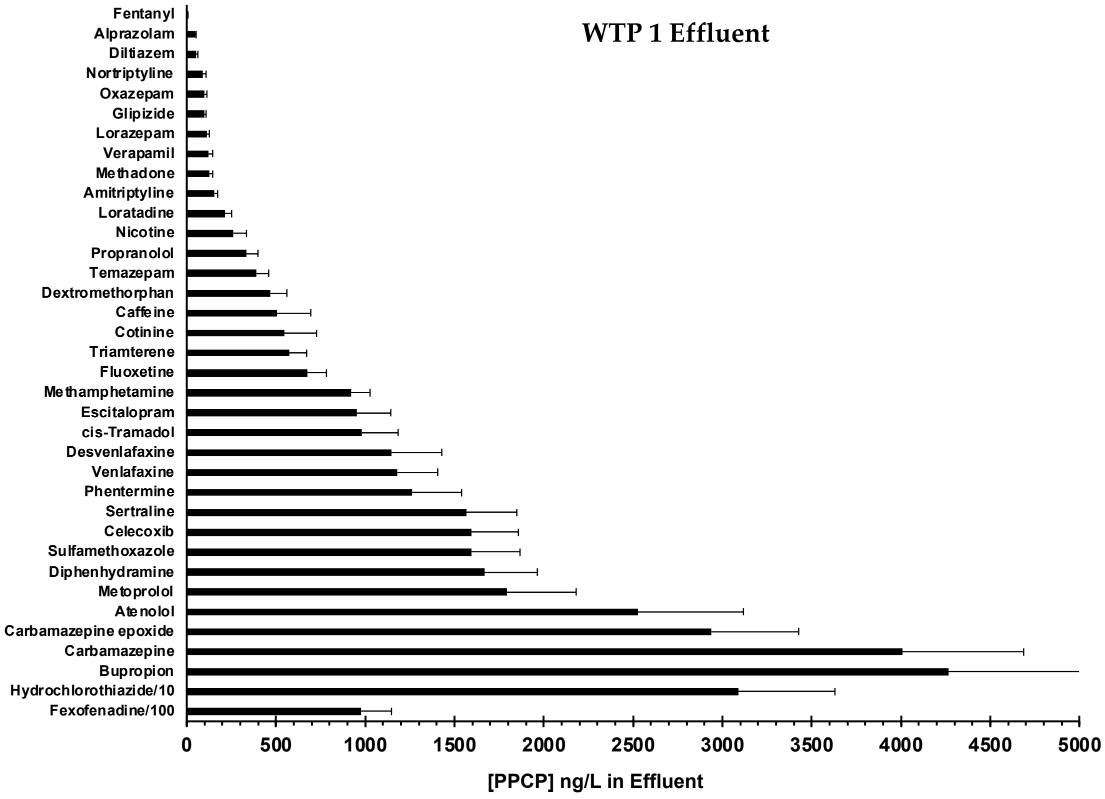

Figure S4. Mean concentrations of PPCPs in effluent from WTP 1 (N=3). Error bar is 1 SD of triplicate measurements.

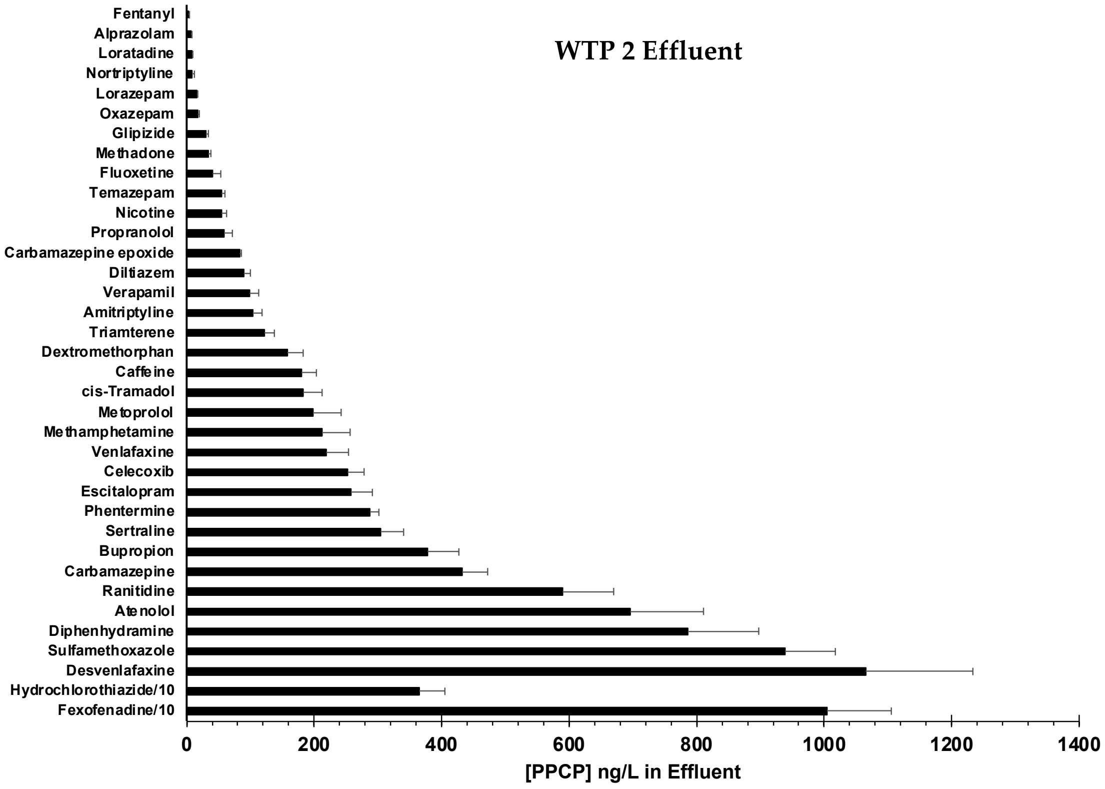

Figure S5. Mean concentrations of PPCPs in effluent from WTP 2 (N=3). Error bar is 1 SD of triplicate measurements.

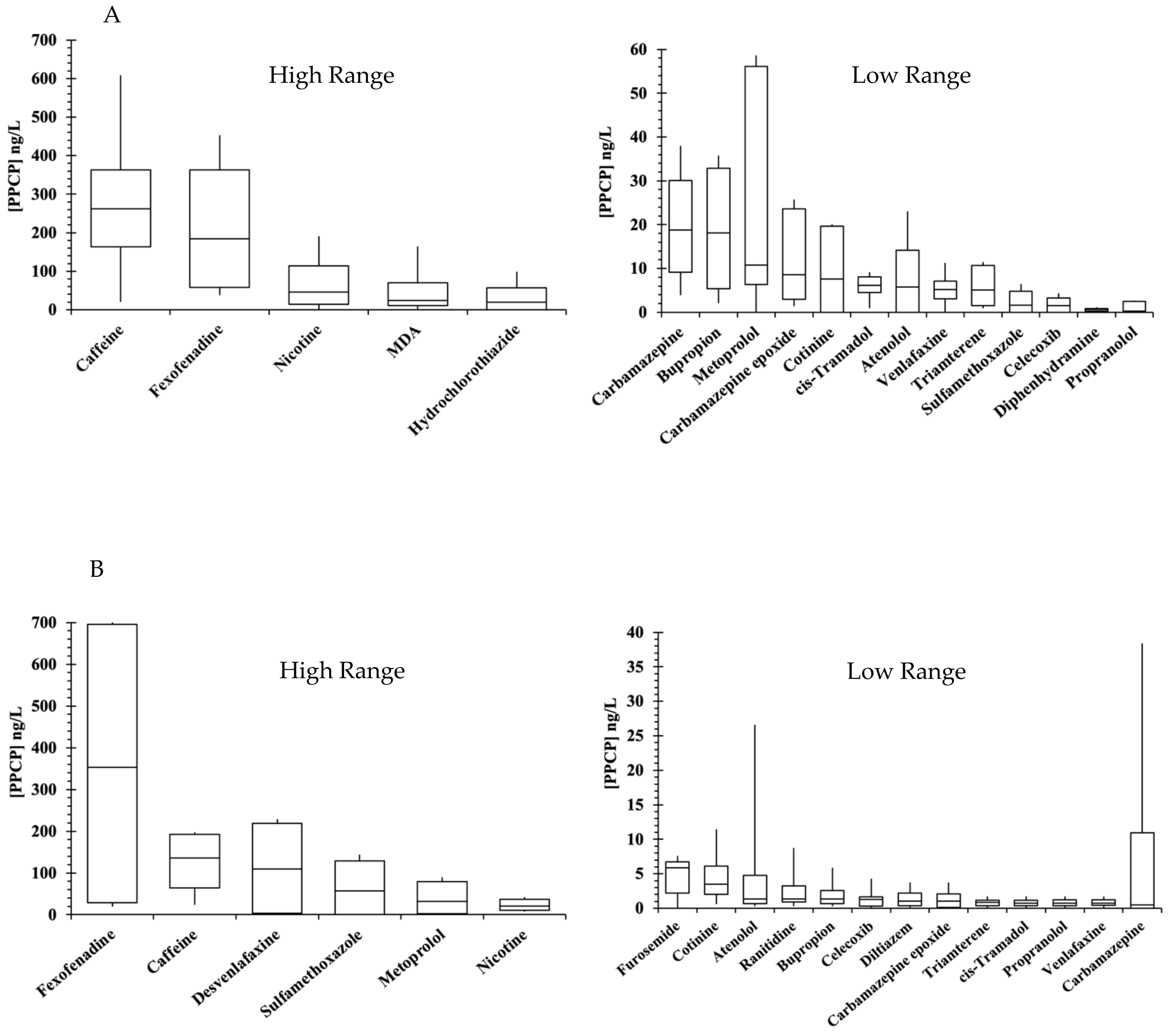

Figure S6. Box plot of individual PPCP concentrations at Four Mile Run (site 3) near WTP 1 (A) and individual PPCP concentrations at Upper Hunting Creek (site 6) near WTP 2 (B). (Boxes represent 25th and 75th percentiles and whiskers 5th and 95th percentiles.) PPCPs are separated into high range and low range concentrations.

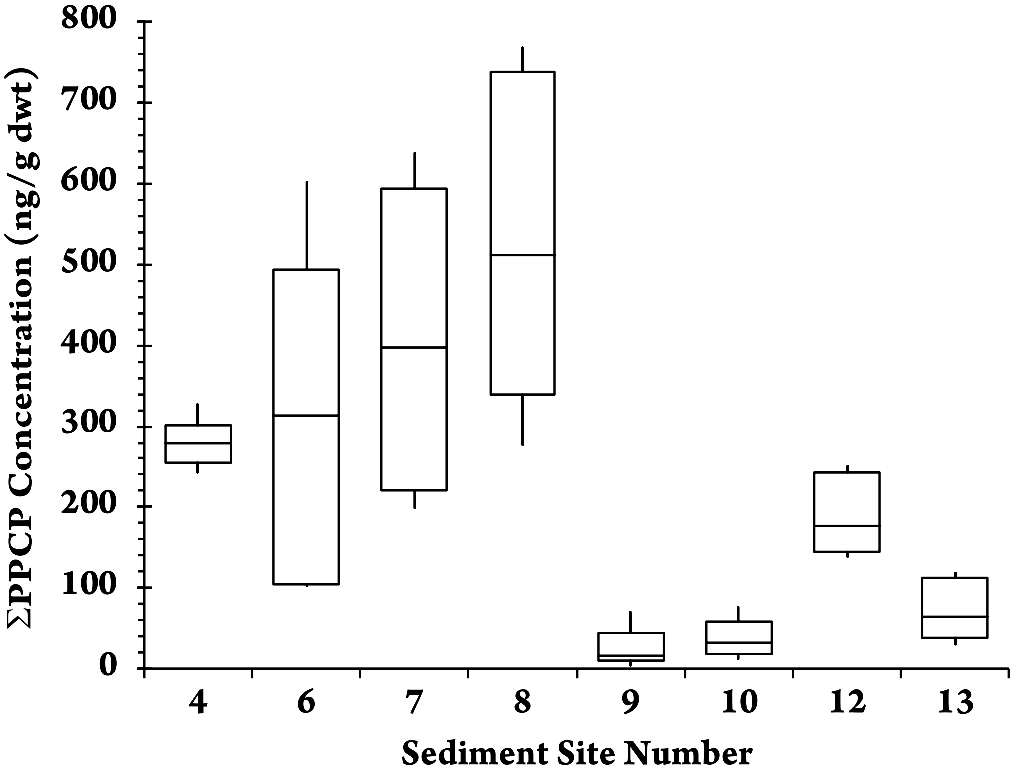

Figure S7. Box plot Σ_{39s}PPCP sediment samples in the tidal freshwater Potomac River. (Boxes are 25th and 75th percentiles; lines are the medians; and whiskers are the 10th and 90th percentiles.).

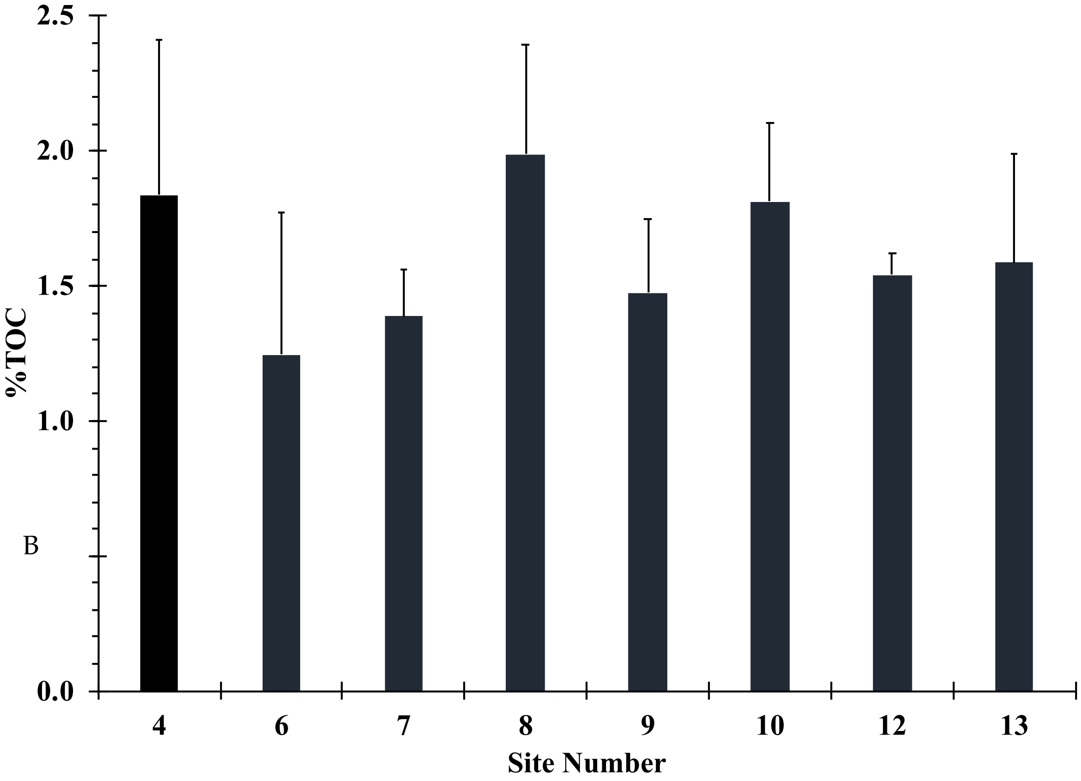

Figure S8. Sediment %total organic carbon (%TOC) content (± 1 SD) at sites in the tidal freshwater Potomac River. Site numbers correspond to Figure 1.
